# Optical Properties of Graphene/MoS_2_ Heterostructure: First Principles Calculations

**DOI:** 10.3390/nano8110962

**Published:** 2018-11-21

**Authors:** Bin Qiu, Xiuwen Zhao, Guichao Hu, Weiwei Yue, Junfeng Ren, Xiaobo Yuan

**Affiliations:** 1 School of Physics and Electronics, Shandong Normal University, Jinan 250014, China; qiubin@stu.sdnu.edu.cn (B.Q.); zhaoxiuwen@stu.sdnu.edu.cn (X.Z.); hgc@sdnu.edu.cn (G.H.); physics_yue@163.com (W.Y.); 2 Institute of Materials and Clean Energy, Shandong Normal University, Jinan 250014, China

**Keywords:** graphene/MoS_2_ heterostructure, optical properties, electronic structure

## Abstract

The electronic structure and the optical properties of Graphene/MoS_2_ heterostructure (GM) are studied based on density functional theory. Compared with single-layer graphene, the bandgap will be opened; however, the bandgap will be reduced significantly when compared with single-layer MoS_2_. Redshifts of the absorption coefficient, refractive index, and the reflectance appear in the GM system; however, blueshift is found for the energy loss spectrum. Electronic structure and optical properties of single-layer graphene and MoS_2_ are changed after they are combined to form the heterostructure, which broadens the extensive developments of two-dimensional materials.

## 1. Introduction

Graphene has been popular among researchers since it was successfully exfoliated by Novoselov et al. in 2004 [1]. Graphene has excellent electrical conductivity [2], excellent mechanical strength [3,4], superior thermal conductivity [5], and high light transmittance in the visible light–infrared area [6]. Graphene has been widely used in applications such as solar cells, lighting, and touch screens [7,8,9,10,11,12,13,14]. However, graphene has been extremely limited in the research and application of some fields because of its zero band gap. One of the methods used to broaden the application of graphene is to form a multilayer structure or heterostructure. Stacking different two-dimensional materials together can form a double-layer or even multi-layer artificial material that is maintained by van der Waals interactions. Such materials are known as van der Waals heterojunctions. Surprising physical properties can be obtained by stacking two-dimensional materials of different properties together. The almost infinitely rich possibilities make the van der Waals heterojunction even more important than the two-dimensional material itself [15,16,17,18]. The large surface area, high chemical resistance, high stability, and good electrical conductivity of graphene indicate that graphene sheets are promising as substrates for improving the electrochemical and electrocatalytic properties of metal oxides and metal sulfides. Properties have already been studied in the heterostructure of Ni(OH)_2_/graphene [19] and SnO_2_/graphene [20], which indicates that the heterostructure of graphene also has great research prospects. On the other hand, heterostructures based on graphene and other two-dimensional materials, such as MoS_2_, will change their electronic structure and other properties, which has attracted people’s attention.

MoS_2_ is one of the transition metal dichalcogenides (TMDs). MoS_2_ can appear in two-dimensional or three-dimensional forms. The direct band gap will be about 1.8 eV [21,22] when MoS_2_ appears as a single-layer two-dimensional material, which makes it a very good semiconductor material. Monolayers of MoS_2_ have many excellent properties, such as high electron mobility, low dimensionality, smooth atomic sheet [21,23], and outstanding mechanical properties [24]. Monolayers of MoS_2_ have been successfully prepared due to their extraordinary properties [25] and have been extensively studied [21,26,27,28,29,30,31]. Furthermore, the heterostructure of graphene/MoS_2_ opens up possibilities for many applications. For example, Ma et al. [32] systematically investigated the electronic and magnetic properties of perfect, vacancy-doped, and nonmetal elements (H, B, C, N, O, and F) adsorbed MoSe_2_, MoTe_2_, and WS_2_ monolayers by means of first-principles calculations. In 2011, Chang et al. [33,34] successfully synthesized layered graphene or graphene nanosheet/MoS_2_ composites by an L-cysteine-assisted solution-phase methodand the obtained composites showed three-dimensional architecture and excellent electrochemical performances which can act as anode materials for Li-ion batteries. Soon Li et al. [35] developed a selective solvothermal synthesis of MoS_2_ nanoparticles on reduced graphene oxide (RGO) sheets and the MoS_2_/RGO hybrid exhibited superior electrocatalytic activity in the hydrogen evolution reaction. Coleman et al. [36] showed that hybrid dispersions or composites could be prepared by blending MoS_2_ with suspensions of graphene or polymer solutions. A recent study reported the catalytic activity of MoS_2_/graphene dots for an oxygen evolution reaction [37]. The above results proved that the heterostructures of GM are useful in applications ranging from electronics to energy storage.

There is still a lack of research of optical properties in GM heterostructures up to now. The heterogeneous structure of graphene has bright prospects of applications and the direct bandgap electronic structure of MoS_2_ is an essential property for many optical applications; so, in this paper, we explore the optical properties of GM based on density functional calculations. The structure of this paper is as follows: Section 2 gives the theoretical calculation method, Section 3 gives the result analysis, and Section 4 gives the conclusion.

## 2. Methods

**Figure 1:**
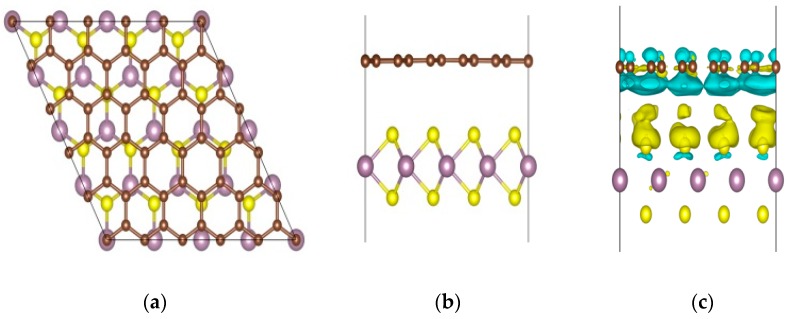
Top (**a**) and side (**b**) views of the Graphene/MoS_2_ (GM) heterostructure. (**c**) The differential charge density distributions of GM. Gray, purple, and yellow atoms represent C, Mo, and S atoms, respectively. Blue means loss electrons and yellow means gain electrons.

The DFT calculations we used are performed by the VASP (Vienna ab-initio Simulation Package) software package [38,39]. The lattice constant of the MoS_2_ monolayer is 3.16Å, and the lattice constant of pure graphene is 2.47Å, so the supercell of MoS_2_ we used was 4*4*1, and the supercell of graphene was 5*5*1. The lattice mismatch ratio of the system was about 2.29%. We stacked monolayer graphene and monolayer MoS_2_ to form the heterostructure of GM, which is shown in Figure 1. In order to reduce the interaction between the periodic structures in the vertical direction when constructing the model, a 20Å vacuum is added. In the theoretical calculations, we use the projector-augmented wave (PAW) [40,41] method to describe the interaction between ions and electrons. At the same time, the exchange-correlation potential is selected based on the Generalized Gradient Approximation (GGA [42]) in terms of the Perdew–Burke–Ernzerhof (PBE [42]) functional, which is often used to calculate the molecular adsorption at the electrode surface. The cutting power of the plane wave is set to 500 eV. When the structure relaxes, the convergence precision of each interatomic force is 0.02 eV/Å, and the self-consistent convergence energy is not higher than 10^−4^ eV. The Brillouin zone was summed according to the 9×9×1 Monkhorst–Pack characteristic K point. Based on the above conditions, the calculated distance between graphene and MoS_2_ is 3.64Å. Then, the electronic structure and the optical properties of the heterostructures are calculated. Van der Waals interactions are included in the calculations.

The optical properties can be modeled by the dielectric constant of the system. We use the superposition of Lorentz oscillators to model the complex dielectric function ε(ω) = ε_1_(ω) + iε_2_(ω) of the heterostructure, which is a function of photon energy. Generally speaking, the dielectric constant is the real part of the complex permittivity, ε_1_(ω). The dielectric constant is caused by various kinds of displacement polarization inside the material and represents the energy storage term of the material. The imaginary part of the complex permittivity, ε_2_(ω), is related to the absorption (loss or gain) of the material. The steering polarization can not keep up with the various relaxation polarizations caused by the change of the external high-frequency electric field, and represents the loss term of the material. The formula of ε_2_(ω) is as follows:

(1) $$\varepsilon_{2}\left(\omega \right)=\frac{4\pi^{2}e^{2}}{\Omega }\underset{q\to 0}{\lim}\frac{1}{q^{2}}\underset{c,v,k}{\sum }2w_{k}\delta \left(\in_{ck}-\in_{vk}-w\right)\times \langle u_{ck}+e_{\alpha q}|u_{vk}\rangle \langle u_{ck}+e_{\beta q}|u_{vk}\rangle^{*}$$

The real part ε_1_(ω) of the dielectric function can be obtained by using the Kramers–Kroing relation,

(2) $$\;\varepsilon_{1}\left(\omega \right)=1+\frac{2}{\pi }P\int_{0}^{\infty }\frac{\varepsilon_{2}^{\alpha \beta }\left(w^{\prime }\right)w^{\prime }}{w^{\prime 2}-w^{2}+i\eta }d\omega^{\prime }\;$$

Other optical constants can also be obtained from the dielectric function. For example, the absorption coefficient α(ω), refractive index n(ω), reflectance R(ω), and energy loss spectrum L(ω) can all be derived by ε_1_(ω) and ε_2_(ω). The formulas are:

(3) $$\;\alpha \left(\omega \right)=\frac{\sqrt{2}\omega }{c}{\left\{{\left[\varepsilon_{1}^{2}\left(\omega \right)+\varepsilon_{2}^{2}\left(\omega \right)\right]}^{\frac{1}{2}}-\varepsilon_{1}\left(\omega \right)\right\}}^{\frac{1}{2}}\;$$

(4) $$\;n\left(\omega \right)=\frac{1}{\sqrt{2}}{\left\{{\left[\varepsilon_{1}^{2}\left(\omega \right)+\varepsilon_{2}^{2}\left(\omega \right)\right]}^{\frac{1}{2}}+\varepsilon_{1}\left(\omega \right)\right\}}^{\frac{1}{2}}\;$$

(5) $$\;R\left(\omega \right)={\left|\frac{\sqrt{\varepsilon_{1}\left(\omega \right)+i\varepsilon_{2}\left(\omega \right)}-1}{\sqrt{\varepsilon_{1}\left(\omega \right)+i\varepsilon_{2}\left(\omega \right)}+1}\right|}^{2}\;$$

(6) $$\;L\left(\omega \right)=\frac{\varepsilon_{2}\left(\omega \right)}{\varepsilon_{1}^{2}\left(\omega \right)+\varepsilon_{2}^{2}\left(\omega \right)}\;$$

## 3. Results and Discussion

**Figure 2:**
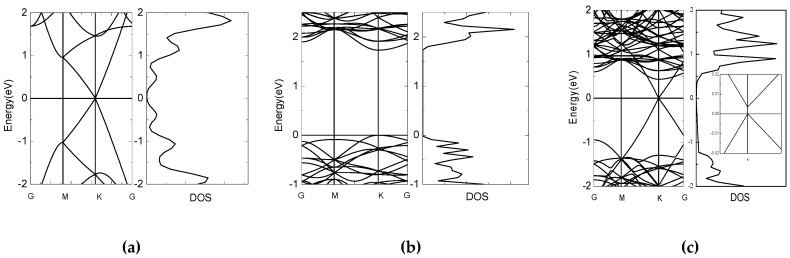
Band structure and density of states (DOS) of graphene (**a**), MoS_2_ (**b**) and the GM heterostructure (**c**), respectively. The embedded figure in (**c**) shows the zoom in of the band structure near Fermi Energy.

In order to illustrate the similarities and the differences of the graphene monolayer, MoS_2_ monolayer, and the GM heterostructure, we first calculate the electronic structures of the three systems. The energy band structures and the electronic density of states (DOS) for the three systems are shown in Figure 2. It can be found that our calculated curves are well matched with the results of previous calculations [43,44]. As shown in Figure 2, graphene is a zero bandgap material and MoS_2_ is a material with a band gap of 1.73 eV. After they are stacked together to form the GM structure, as shown in Figure 1, the band gap is 3.49 meV for GM heterostructures, which can be obtained from the embedded figure in Figure 2c. Based on the interlayer interactions between G and M, there will be a change in the on-site energy of atoms in the G layer, so the band gap opens [44,45]. The upward shift of the Dirac point of graphene with respect to the Fermi level indicates that holes are donated by the MoS_2_ monolayer, which can be confirmed by the charge transfer between graphene and MoS_2_ after stacking. Figure 1c gives the differential charge density distributions, blue means loss electrons and yellow means gain electrons. It is clear from the figure that holes in G are donated by M monolayer after the stacking. From Figure 2 we can clearly see that after the heterostructure is formed, the electronic structure changes greatly. Therefore, we speculate that the formation of the GM heterostructure will influence the optical properties compared with single-layer graphene or MoS_2_.

**Figure 3:**
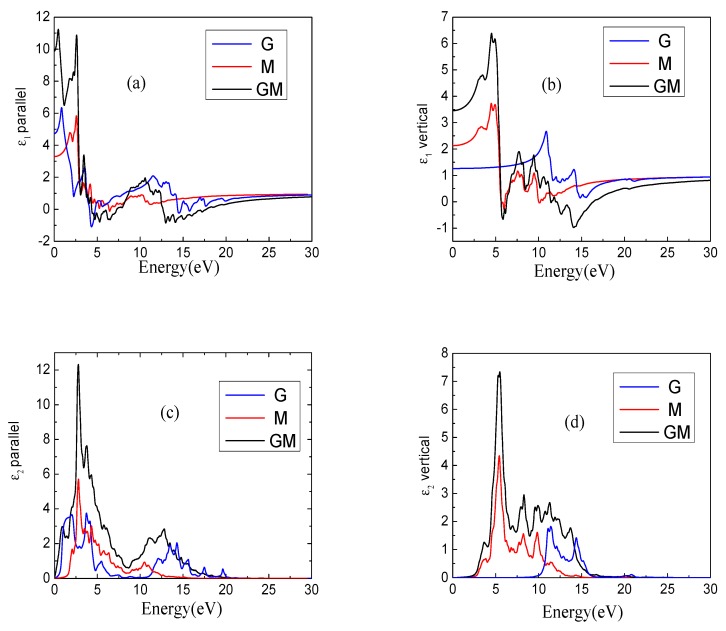
The complex dielectric constants of monolayer graphene (G), monolayer MoS_2_ (M) and GM systems. (**a**,**b**) represent the parallel and vertical components of the real part of the dielectric constant, (**c**,**d**) represent parallel and vertical components of the imaginary part of the dielectric constant, respectively.

The calculated dielectric constants ε(ω) of the monolayer graphene (G), monolayer MoS_2_ (M), and GM heterostructure are shown in Figure 3. Figure 3a shows the parallel direction of the ε_1_(ω). We can clearly see from the figure that the overall trends for all systems are almost identical with only small differences. In fact, people are more interested in the changes that occur in the visible light region. In the visible light region, the value of the ε_1_(ω) is obviously the largest in the GM system, followed by the M system, and finally the G system. Comparing the GM and G system at the low-energy zone, it can be found that the parallel direction of ε_1_(ω) for the two systems not only changes at the maximum values, but also GM has an obvious blueshift of ε_1_(ω) relative to the G system. Figure 3b shows the ε_1_(ω) in the vertical direction and we find similar regularity with those of Figure 3a. Under the same analysis of the three systems in the low-energy region, we find that the most obvious change is a more obvious redshift for the GM system compared with the G system. This is because the GM system is an anisotropic material and the parallel direction of the ε_1_(ω) illustrates differences in the vertical and horizontal directions. Figure 3c, d shows the parallel and vertical directions of the imaginary part of the dielectric constant, respectively. Same properties between the real and the imaginary parts of ε_1_(ω) can be found. The peak value of the dielectric constant of the GM system has been significantly improved compared with G and M and different degrees of redshift or blueshift can also be found.

**Figure 4:**
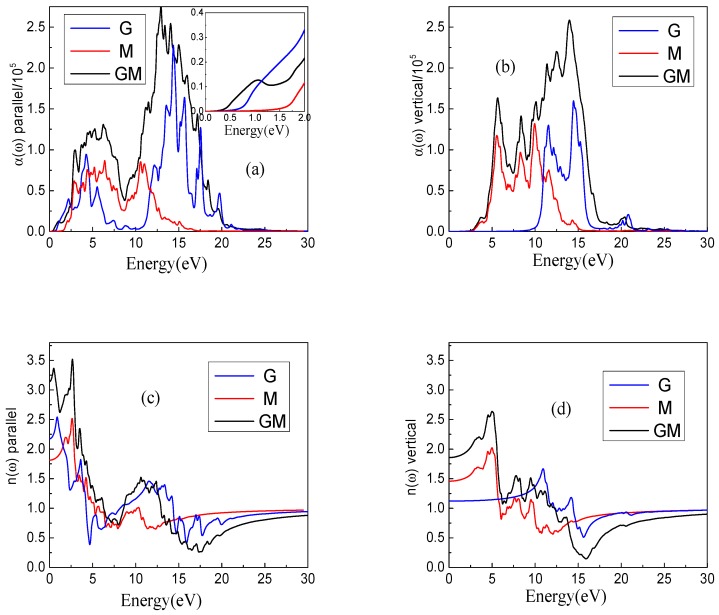
The absorption coefficient α(ω) and the refractive index n(ω) of three systems. (**a**,**b**) represent parallel and vertical components of the absorption coefficient α(ω), (**c**,**d**) represent parallel and vertical components of the refractive index n(ω), respectively.

Figure 4a shows the absorption coefficient α(ω) in the parallel direction. The overall change trend of the GM and M systems are similar, and the only difference is in the peak values. There are obvious differences between the GM and G systems. The α(ω) of the GM system is more volatile than the G system at the peak position. Among the three systems, GM usually has a large α(ω) value in most cases; however, in the visible region, G is slightly larger than that of the GM system. A zoom in the region between 0 and 2 eV of Figure 4a is also embedded. The value of the intersection between the reverse tangent and the x-axis is the optical band gap in the region between 0 and 2 eV in Figure 4a. It can be found from Figure 4a, that the optical band gap of G is about 0.75 eV, and the optical band gap of M is about 1.63 eV. However, the band gaps are around 0.41 eV and 1.40 eV when the system is going from G and M to GM. It is well known that a photoelectron can be excited with less energy when the optical band gap is small. The optical band gap of GM is significantly reduced, which indicates that we can use a lower energy to excite a photoelectron in GM compared with the G and M systems. The vertical direction of α(ω) is given in Figure 4b. The overall change trend of the vertical direction, α(ω), has a similar regularity compared with the parallel direction. The obvious difference is that the GM system has a large redshift in the vertical direction compared with the G system. The α(ω) is greatly improved for the GM system compared with the G and M systems, so the GM system is indeed superior to the G and M systems in terms of absorption properties.

The parallel direction and vertical direction of the refractive index n(ω) are given in Figure 4c,d, respectively. According to the formula for calculating the refractive index, i.e., Equation (4), we can see that the refractive index is essentially related to the real and the imaginary parts of the dielectric constant. By comparing the dielectric constant of Figure 3 and the refractive index image of Figure 4, it can be found that the change trends of Figure 3a,b are similar with those in Figure 4c,d, which means that the effects of the real part of the dielectric constant on the refractive index play the leading role. We found that the n(ω), especially in the visible light range, has a large value for the GM system. The heat preservation characteristics will be good if the material has a big refractive index. This property can be applied to materials that require constant temperature conditions.

**Figure 5:**
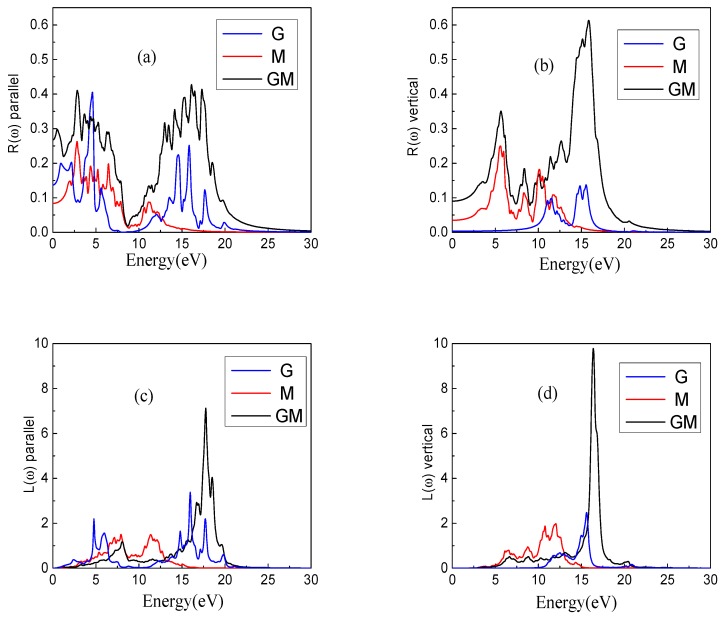
The reflectance R(ω) and the energy loss spectrum L(ω) of three systems. (**a**,**b**) represent parallel and vertical components of the reflectance R(ω), (**c**,**d**) represent parallel and vertical components of the energy loss spectrum L(ω), respectively.

The parallel and vertical directions of the reflectance R(ω) are given in Figure 5a,b, respectively. For parallel directions, the GM system is significantly higher than those of the G and M systems, especially in the visible region. It is obvious that the GM system has a certain redshift relative to the G system, and this phenomenon is also reflected in the vertical direction. In the visible light region, the value of the GM system is also higher than those of the other two systems.

The energy loss spectra L(ω) are given in Figure 5c,d. In the parallel direction, L(ω) of the GM in the low-energy region is significantly less than those of the other two systems. Especially for the G system, the maximum energy loss in the low-energy zone reaches 2, while the GM system is around 0.3. As the energy increases, energy losses also increase. The energy loss of the GM system is concentrated inthe range of 15–20 eV, however for the G and M systems, the energy losses are concentrated in the range of 5–20 eV and they span a large energy extent. In the vertical direction, the energy losses of the three systems in the low-energy region are almost zero, indicating that the loss of power in the vertical direction is small in the low-energy region. The energy loss of the GM system is almost concentrated between 15 eV and 18 eV, while the energy of the G and the M system are lost relatively evenly between 5 eV and 15 eV, which means that the ability to control the energy loss of the GM system is the best. In addition, the GM system is relatively blueshifted for both horizontal and vertical energy loss compared with the G and M systems.

## 4. Conclusions

In this article, we mainly discuss the electronic structure and the optical properties of GM heterostructures from the first principles calculations. Based on the DFT theory, dielectric constant, ε(ω) absorption coefficient α(ω), refractive index n(ω), reflectivity R(ω), and energy loss spectrum L(ω) of the systems are calculated. It is found that there is indeed a clear improvement of the optical properties for the GM system comparedto the G and M systems. The band gap and the dielectric constants become large for the GM system and there are redshifts for the absorption coefficient, refractive index, and the reflectance. A blueshift is found for the energy loss spectrum in the GM system. All of the above results show that, due to the formation of the heterojunctions, the optical properties of the GM system have been significantly improved compared with the single layers, which deliversa more effective way to use two-dimensional materials in optical applications.
